# Small Pox

**Published:** 1855-02

**Authors:** 


### EDITORIAL.

*Small Pox.*—During the first week in January, cases of small pox occurred in our city, in such numbers, and simultaneously in so many different localities, and among so many classes of people, as to leave but little doubt that they depended, in part at least, on a true epidemic influence. And judging from the character of the cases which occurred in those not protected in any degree by vaccination, we think that without the influence of the latter on the great mass of the people, we should be to-day in the midst of as loathsome a pestilence as ever afflicted the cities of Europe during the middle ages. It was this disease which caused the death of the two medical students, whose early departure from the scenes of life, is appropriately noticed in the preamble and resolutions which follow.

The extent of its prevalence, will be more fully noticed in our next number.

